# Interrelationship Between Socioeconomic Status, Depression, and Neuropathy in People with Diabetes: A Cross-Sectional Study

**DOI:** 10.3390/jcm15093215

**Published:** 2026-04-23

**Authors:** Raabya Pasha, Gifty Quartey, Alise Kalteniece, Catharina Faber, Giuseppe Lauria, Andrew Marshall, Shazli Azmi, Rayaz A. Malik, Handrean Soran, Maryam Ferdousi

**Affiliations:** 1 Division of Cardiovascular Sciences, Faculty of Biology, Medicine and Health, The University of Manchester, Manchester M13 9PT, UKgiftyequartey@gmail.com (G.Q.);; 2 Manchester University NHS Foundation Trust, Manchester M13 9WL, UK; 3 NIHR/Wellcome Trust Clinical Research Facility, Manchester M13 9WL, UK; 4 Department of Neurology, School of Mental Health and Neuroscience, Maastricht University Medical Centre, 6229 HX Maastricht, The Netherlands; 5 Neuroalgology Unit, IRCCS Foundation, “Carlo Besta” Neurological Institute, 20133 Milan, Italy; 6 Department of Biomedical and Clinical Sciences, “Luigi Sacco”, University of Milan, 20157 Milan, Italy; 7 Institute of Cardiovascular Sciences, Cardiac Centre, Faculty of Medical and Human Sciences, University of Manchester, Manchester M13 9PT, UK; andrew.marshall@liverpool.ac.uk (A.M.); ram2045@qatar-med.cornell.edu (R.A.M.); 8 Department of Diabetes, Endocrinology and Metabolism, Manchester University NHS Foundation Trust, Manchester M13 9WL, UK; 9 Department of Medicine, Weill Cornell Medicine-Qatar, Doha 24144, Qatar

**Keywords:** depression, diabetes, neuropathy, questionnaires, socioeconomic status

## Abstract

**Background/Objectives**: The co-occurrence of diabetic peripheral neuropathy and depression increases the symptom burden and risk of long-term complications. **Methods**: This cross-sectional study enrolled 131 patients with type 1 (age: 58.47 years; duration of diabetes: 35.61 years) and type 2 diabetes (age: 63.60 years; duration of diabetes: 11.49 years). All patients underwent assessment of socioeconomic status and evaluation using the Hospital Anxiety and Depression Scale, the Mental Component Score of the Short Form Healthy Survey Questionnaire, neuropathy disability score, nerve conduction studies, corneal confocal microscopy and intraepidermal nerve fibre density (IENFD) assessment. **Results**: The prevalence of foot pain (45% vs. 23.9%, *p* = 0.019), tingling (56.7% vs. 32.9%, *p* = 0.013), weakness (35% vs. 9.9%, *p* < 0.001), ataxia (40% vs. 16.9%, *p* = 0.001), and upper limb symptoms (45% vs. 19.7%, *p* = 0.001) were statistically significantly higher, while cold perception threshold (22.50 ± 8.47 vs. 26.34 ± 3.08, *p* = 0.007), corneal nerve fibre density (20.49 ± 7.55 vs. 24.16 ± 5.68, *p* = 0.002) and length (20.06 ± 6.98 vs. 22.95 ± 6.22, *p* = 0.014) were statistically significantly lower, but no differences in nerve conduction studies or IENFD were observed in patients with depression compared to patients without depression. Furthermore, patients with depression were from a lower socioeconomic class (51.7% vs. 21.1%, *p* < 0.001), had lower educational attainment (37.9% vs. 12.9%, *p* < 0.001), had lower income < £37,000 (29.3% vs. 11.4%, *p* = 0.010) and lived in areas of high deprivation (62.1% vs. 31.4%, *p* < 0.001). **Conclusions**: Comorbid depression in people with diabetes was linked to increased socioeconomic deprivation and a greater prevalence of neuropathic symptoms and small fibre pathology.

## 1. Introduction

Diabetic peripheral neuropathy (DPN) affects over half of individuals with diabetes [1,2] and is characterised by pain, numbness, weakness, and impaired balance and mobility [3], with a decline in quality of life [4]. The co-occurrence of DPN and depression may exacerbate symptom burden, disrupt self-care, and further decrease quality of life [5]. Neuropathic pain and disability may increase vulnerability to depression [6].

Depression is a global public health priority [7] associated with a broad spectrum of cognitive and physiological disturbances [8] which exerts a substantial toll on quality of life [9]. A meta-analysis by de Groot et al. showed that depression statistically significantly increases the risk of diabetes complications, and individuals with painful neuropathy are twice as likely to report depressive symptoms. However, the directionality and underlying mechanisms of this association remain debated [10].

Socioeconomic status (SES) introduces an additional multifaceted construct encompassing income, education, employment, and social environment, which is strongly linked with poorer health outcomes and increased psychological morbidity [11]. Indeed, individuals from socioeconomically deprived backgrounds are disproportionately affected by both DPN and depression [12,13,14], driven by limited access to preventive care and social support, with lower health literacy and chronic stress [15].

Corneal confocal microscopy (CCM) is a surrogate marker of small nerve fibre damage in DPN, and CCM-derived nerve parameters are lower in patients with painful compared with painless DPN [16,17]. Furthermore, Kalteniece et al. (2022) showed that CCM-derived nerve metrics were associated with the severity of painful DPN [18].

The relationships between socioeconomic disadvantages, depression, metabolic control, and diabetic neuropathy are likely to be complex and interrelated. Lower socioeconomic status has consistently been associated with both increased prevalence of depression and poorer glycaemic control in individuals with diabetes [9,19,20,21]. Depression has been linked to suboptimal self-management behaviours and higher glycated haemoglobin A1c (HbA1c) levels [22,23], while hyperglycaemia remains a key risk factor for the development and progression of neuropathy [24]. At the same time, neuropathic pain and functional impairment may contribute to psychological distress, suggesting a potentially bidirectional relationship between depression and neuropathy [25,26,27]. Furthermore, socioeconomic deprivation has been independently associated with symptomatic diabetic neuropathy [12,13]. Taken together, these findings suggest that socioeconomic and psychological factors may coexist with, and potentially influence, neuropathic burden in diabetes.

However, the temporal relationships between these variables remain uncertain, and cross-sectional analyses cannot establish causality. Although socioeconomic status, depression, and diabetic peripheral neuropathy have been independently studied, there remains a lack of integrated analysis examining how socioeconomic disadvantage may relate to the association between depression and neuropathy, particularly in the context of small fibre damage.

Although socioeconomic status, depression, and DPN have been independently investigated, there remains a limited understanding of how these factors interact, particularly in relation to objective measures of small fibre damage. In this cross-sectional study, we aimed to investigate the interrelationships between depression, DPN, and socioeconomic status in individuals with diabetes using a comprehensive assessment including clinical neuropathy scores, nerve conduction studies, CCM, and intraepidermal nerve fibre density (IENFD). This integrated approach allows the evaluation of both psychosocial and biological contributors to neuropathic burden in diabetes.

## 2. Materials and Methods

### 2.1. Study Design and Participants

This cross-sectional observational study recruited 131 patients with type 1 (*n* = 39, age: 58.48 years; duration of diabetes: 35.62 years) or type 2 diabetes (*n* = 92, age: 63.60 years; duration of diabetes: 11.49 years) at Manchester University NHS Foundation Trust. Eligible patients were identified during routine clinical appointments and introduced to the study by their treating clinician. Those who expressed interest were subsequently contacted by the research team, and written informed consent was obtained prior to participation.

Participants were excluded if they had a history of malignancy, vitamin B12 or folate deficiency, chronic renal or hepatic impairment, connective tissue disease, active systemic or infectious disease, neuropathy attributable to causes other than diabetes, an active diabetic foot ulcer, previous corneal trauma, systemic corneal pathology, or current contact lens use.

The study was approved by the NRES Committee North West—Greater Manchester East (Approval Code: 14/NW/0093; Approval Date: 5 March 2014) and was conducted in accordance with the principles of the Declaration of Helsinki.

#### 2.1.1. Demographic and Clinical Neuropathy Assessment

Body mass index (BMI), blood pressure, lipid profile and HbA1c were measured in each participant. The neuropathy disability score (NDS) assesses vibration perception, temperature sensation, pinprick perception and the Achilles tendon reflex. Each test is scored as either present ‘0’ or absent/abnormal ‘1’, except for Achilles reflex, where reinforcement is scored ‘1’ and absent/abnormal as ‘2’ to give a total score out of 10 [28]. The NDS is used to further categorise the severity of neuropathy in patients: no neuropathy (NDS = 0–2), mild (NDS = 3–5) and moderate to severe neuropathy (NDS = 6–10) [28].

Vibration perception threshold (VPT) was quantified using a neurothesiometer [29] device (Horwell, Scientific Laboratory Supplies, Wilford, Nottingham, UK) on both feet. Deep breathing–heart rate variability (DB-HRV) was measured using the ZOE PPM3 autonomic nervous system monitoring device (Physio PS, Inc., Londonderry NH, USA). The Medoc TSA-II NeuroSensory Analyzer (Ramat Yishai, Israel) was used to assess cold (CPT) and warm (WPT) perception thresholds. IENFD was assessed in 3 mm punch skin biopsies obtained from the distal leg, using standard immunohistochemical staining techniques [17].

Neuropathic pain was assessed using a visual analogue scale (VAS), in which participants rated their average neuropathic pain intensity over the past week on a 10-point scale (0 = no pain, 10 = worst imaginable pain), providing a continuous measure of pain severity [30].

The modified Toronto Clinical Neuropathy Score was used to evaluate the clinical signs and symptoms of diabetic peripheral neuropathy. The score is based on 6 symptom scores (presence or absence of foot pain, numbness, tingling, weakness, imbalance, and upper limb symptoms), reflex scores (bilateral knee and ankle reflexes, each graded as absent, reduced, or normal), and 5 physical examination scores (presence or absence of pinprick, temperature, light touch, vibration, and position sense) [31].

The Dantec “Keypoint” system (Dantec Dynamics Ltd., Bristol, UK) was used to conduct nerve conduction studies (NCS) while a DISA temperature regulator was used to ensure that the temperature of the limb stayed between 32 and 35 °C. A consultant neurophysiologist assessed the sural nerve latency, sural nerve amplitude and sural nerve velocity.

DPN was diagnosed based on the Toronto Consensus, according to the presence of ≥6 symptoms [31] or signs of neuropathy (NDS > 2) and an abnormal sural nerve conduction velocity (<40 m/s) [32]. Painful DPN was defined by characteristic neuropathic pain [33] and a VAS score > 4.

#### 2.1.2. Corneal Confocal Microscopy

CCM was performed using the Heidelberg Retinal Tomograph III with the Rostock Cornea Module (Heidelberg Engineering GmBH, Heidelberg, Germany). Six high-quality images of the central sub-basal nerve plexus (three per eye) were selected and analysed using CCMetrics software (Version 3, June 2015, University of Manchester), following a validated protocol [34]. CCM image analysis was performed in a masked manner. Graders were blinded to participants’ depression and SES. Three parameters were quantified: corneal nerve fibre density (CNFD) (no./mm^2^), corneal nerve branch density (CNBD) (no./mm^2^) and corneal nerve fibre length (CNFL) (mm/mm^2^).

#### 2.1.3. Assessment of Depression

The HADS questionnaire, which measures the lack of positive affect and pleasure coming from carrying out daily tasks, was used, with a higher score indicating severe symptomatology. Patients receive a score of 0–21 for anxiety and 0–21 for depression—a score of 0–7 on either subscale is normal, 8–10 is suggestive of the presence of the respective mood state and ≥11 indicates the presence of a mood disorder [35].

The SF-36 was also undertaken, in which questions 5, 6, 9 and 10 assess vitality, social functionality, and role-emotional and mental health, which make up the mental component score (MCS), with a score of ≤42 indicative of depression [36].

Depressive symptomatology was defined by a HADS depression score ≥ 11 and/or an SF-36 MCS ≤ 42, thereby capturing both clinically statistically significant depressive symptoms and impaired mental health-related quality of life. Participants were subsequently stratified into those with depression (*n* = 60) and those without (*n* = 71).

#### 2.1.4. Assessment of Socioeconomic Status

Individual-level socioeconomic data were not available, as these are not routinely collected in clinical datasets. Socioeconomic status was therefore derived from postcode-linked area-level indicators using Office for National Statistics (ONS) data and the Index of Multiple Deprivation (IMD), a widely validated measure in UK epidemiological research.

SES was assessed using secondary data from the 2021 government census [37]. Using patients’ postcodes, we extracted area-level indicators, including mean household income (MHI), socioeconomic classification (SEC), IMD and the highest level of qualification (LOQ) achieved based on regional averages [37].

Socioeconomic classification was determined according to occupational status: (L1–L6—administrative and professional roles), (L7—intermediate occupations), (L8–L9—small employers and own-account workers), (L10–L13—lower supervisory and routine occupations), (L14—long-term unemployment), and (L15—full-time education), with a lower classification number reflecting a higher socioeconomic status and individuals with a score of 13 and above meeting the deprivation criteria [37].

Educational attainment was grouped into three categories—no qualifications, level 1 to 3 (GCSEs or A-levels), and level 4 or above (university degrees or equivalent) [37]—and individuals without qualifications met the criteria for educational deprivation [37]. Household income was measured based on the mean gross income for each patient’s postcode area, with those who earned £37,000 or less being classified as having income deprivation [37].

The IMD is derived from the following: income deprivation, employment deprivation, education, skills and training, health deprivation and disability, crime, barriers to housing and services house domain, living environment deprivation, income deprivation affecting children and older people index [38]. The collective scores ranged from 0 to 1.0, with 0–0.4 indicating low deprivation, 0.4–0.7 moderate deprivation and 0.7–1.0 high deprivation [37]. Individuals who scored above 0.7 met the criteria for multiple deprivation [38].

#### 2.1.5. Statistical Analysis

Statistical analysis was conducted using SPSS (Version 28.0.1.0, IBM Corporation, New York, NY, USA). The Shapiro–Wilk normality test was used to determine the normality of the data. Data are presented as mean ± standard deviation (SD) for parametric variables. Differences in continuous variables between groups were assessed using an independent samples *t*-test (non-parametric: Mann–Whitney U test) for two groups, and one-way ANOVA (non-parametric: Kruskal–Wallis test) for more than two groups. To account for multiple testing in the comparison of individual neuropathic symptom domains, *p*-values were adjusted using the Benjamini–Hochberg false discovery rate (FDR) procedure, with an FDR threshold of 0.05.

## 3. Results

### 3.1. Demographic Data

A total of 131 patients with diabetes were categorised into two groups: with depression (*n* = 60) and without depression (*n* = 71). Age (*p* = 0.060) and duration of diabetes (*p* = 0.872) did not differ; however, BMI (32.85 ± 9.72 vs. 29.07 ± 4.76 kg/m^2^, *p* = 0.004) and HbA1c (59.74 ± 12.63 vs. 53.22 ± 15.07 mmol/mol, *p* = 0.001) were statistically significantly higher in those with depression compared with patients without depression (Table 1).

The prevalence of DPN was not statistically significantly different (26.7% vs. 26.8%, *p* = 0.990), but the prevalence of painful DPN (23.3 vs. 7%, *p* < 0.001) was statistically significantly higher in patients with depression compared with those without. Patients with depression were statistically significantly prescribed more neuropathic pain medication compared with patients without depression (38.3 vs. 11.3%, *p* < 0.001).

### 3.2. Neuropathy Assessments

**Figure 1:**
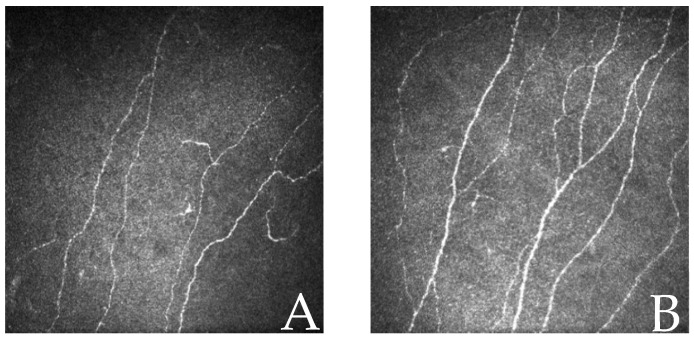
CCM images of patients with diabetes: (**A**) with depression and (**B**) without depression.

Patients with depression had a statistically significantly lower CNFD (20.49 ± 7.55 vs. 24.16 ± 5.68 no./mm^2^, *p* = 0.002) and CNFL (20.06 ± 6.98 vs. 22.95 ± 6.22 mm/mm^2^, *p* = 0.014) compared with patients without depression (Table 2, Figure 1).

CPT was statistically significantly lower in patients with depression compared with patients without depression (22.50 ± 8.47 vs. 26.34 ± 3.08 °C, *p* = 0.007).

No statistically significant difference was found in CNBD, IENFD, NDS, any of the nerve conduction velocity parameters (sural nerve latency, nerve amplitude and velocity), VPT and DB-HRV. Sensory score and WPT were also not statistically significantly different between the two groups (i.e., with depression and without depression).

### 3.3. Signs and Symptoms

**Figure 2:**
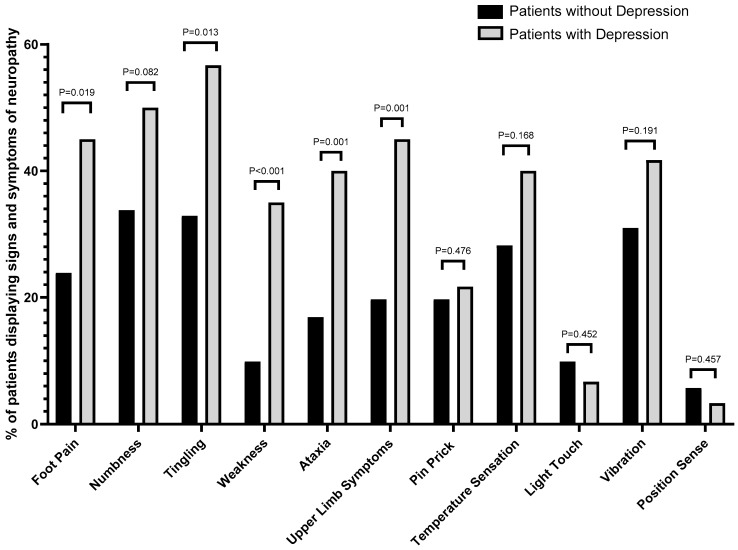
The percentage of patients with and without depression experiencing different signs and symptoms. *p*-values shown are Benjamini–Hochberg FDR-adjusted (11 comparisons, q = 0.05).

The percentage of patients with signs and symptoms of neuropathy was statistically significantly higher in patients with depression (Figure 2). Foot pain (45% vs. 23.9%, *p* = 0.019), tingling (56.7% vs. 32.9%, *p* = 0.013), weakness (35% vs. 9.9%, *p* < 0.001), ataxia (40% vs. 16.9%, *p* = 0.001) and upper limb symptoms (45% vs. 19.7%, *p* = 0.001) were statistically significantly higher in patients with depression compared with patients without depression. Following Benjamini–Hochberg FDR correction across 11 symptom/sign comparisons, five symptom domains remained statistically significant, while numbness did not remain significant after adjustment (raw *p* = 0.045; *p*(FDR) = 0.0825).

### 3.4. Socioeconomic Status

**Figure 3:**
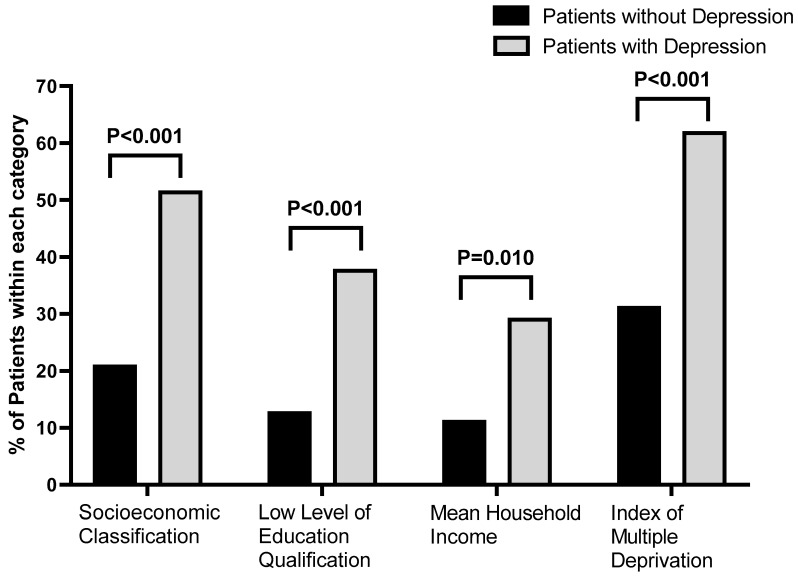
The percentage of patients with and without depression in four categories and the percentage of individuals from both cohorts—with and without depression—meeting the deprivation criteria of each category: socioeconomic classification ≥ 13, low level of qualification ≤ 0, mean household income ≤ £37,000 and Index of Multiple Deprivation ≥ 7.

Patients with depression demonstrated statistically significantly greater socioeconomic disadvantage compared with those without depression (Figure 3). They were more likely to fall into the lower SEC (51.7% vs. 21.1%, *p* < 0.001), have a higher proportion with low educational attainment (37.9% vs. 12.9%, *p* < 0.001), and live in areas with MHI below £37,000 (29.3% vs. 11.4%, *p* = 0.010) and high deprivation according to the IMD (62.1% vs. 31.4%, *p* < 0.001).

### 3.5. Correlations

There were no statistically significant correlations between any of the CCM parameters and socioeconomic categories. VAS had a statistically significant positive correlation with SEC (r = 0.419, *p* < 0.001) and IMD (r = 0.442, *p* < 0.001) and a statistically significant negative correlation with LOQ (r = −0.331, *p* < 0.001) and MHI (r = −0.453, *p* < 0.001) (Appendix A).

The HADS score positively correlated statistically significantly with SEC (r = 0.421, *p* < 0.001) and IMD (r = 0.432, *p* < 0.001) and had a statistically significant negative correlation with LOQ (r = −0.322, *p* < 0.001) and MHI (r = −0.462, *p* < 0.001).

The SF-36 score had a statistically significant negative correlation with SEC (r = −0.337, *p* < 0.001) and IMD (r = −0.333, *p* < 0.001) and a statistically significant positive correlation with LOQ (r = 0.310, *p* < 0.001) and MHI (r = 0.252, *p* = 0.004).

The VAS and HADS scores had a statistically significant positive correlation with each other (r = 0.522, *p* < 0.001), and the SF-36 score had a statistically significant negative correlation with the VAS (r = −0.427, *p* < 0.001) and HADS scores (r = −0.670, *p* < 0.001).

VAS had a statistically significant negative correlation with CNFD (r = −0.311, *p* < 0.001), CNBD (r = −0.182, *p* = 0.038), CNFL (r = −0.231, *p* = 0.008), DB-HRV (r = −0.271, *p* = 0.004) and CPT (r = −0.345, *p* < 0.001). VAS had a statistically significant positive correlation with NDS (r = 0.226, *p* = 0.009), WPT (r = 0.316, *p* < 0.001), symptom (r = 0.635, *p* < 0.001) and sensory (r = 0.290, *p* < 0.001) scores and VPT (r = 0.198, *p* = 0.024).

The HADS score was statistically significantly negatively correlated with CNFD (r = −0.229, *p* = 0.009), CNFL (r = −0.191, *p* = 0.029) and CPT (r = −0.266, *p* = 0.002) and statistically significantly and positively correlated with the symptom score (r = 0.551, *p* < 0.001).

The SF-36 score statistically significantly correlated positively with CNFD (r = 0.241, *p* = 0.006), CNBD (r = 0.206, *p* = 0.019) and CNFL (r = 0.257, *p* = 0.003) and negatively with the symptom score (r = −0.515, *p* < 0.001).

HbA1c had a statistically significant negative correlation with SF-36 (r = −0.279, *p* = 0.043) and a statistically significant positive correlation with the VAS score (r = 0.186, *p* = 0.049), SEC (r = 0.250, *p* = 0.008) and IMD (r = 0.211, *p* = 0.027).

## 4. Discussion

Individuals with diabetes and DPN have higher rates of depression [39], which adversely affects patient engagement in essential self-care behaviours, contributing to suboptimal glycaemic control and increasing the risk of diabetes-related complications [22]. Moreover, patients with depression are approximately three times more likely to exhibit poor medication adherence [40]. In the present study, we found that more patients with depression were prescribed analgesic medication compared with patients without depression, and yet patients with depression experienced more severe neuropathic symptoms. While this may reflect differences in pain perception or treatment response, medication adherence was not assessed in this study and therefore no conclusions can be drawn regarding its role. An increase in the prevalence of symptoms of depression has been linked to an increase in the number and severity of diabetes complications [10]. D’Amato et al. reported that depression was twice as prevalent in patients with painful DPN compared with painless DPN [25]. VPT and NDS were significantly associated with a higher prevalence of symptoms of depression, and higher levels of depression were associated with pain and reduced feeling in the foot and unsteadiness [26]. Indeed, in our cohort, foot pain was more prevalent in patients with depression. Painful DPN has been associated with symptoms of depression, with a greater negative impact in patients with more severe pain [27]. Gharaibeh et al. recently reported no association between depression and neuropathic pain, but there was an association with the severity of neuropathy [41].

In the present study, CCM demonstrated reduced CNFD and CNFL in individuals with depression; however, no significant differences were observed in IENFD or nerve conduction studies. While both CCM and IENFD assess small fibre structure with comparable diagnostic efficiency [42], the absence of differences in IENFD in the present study suggests that the observed CCM changes may reflect subtle or early alterations not detected by other measures and should therefore be interpreted cautiously. It is important to note, though, that only 18 participants (*n* = 12 without depression vs. *n* = 6 with depression) undertook the IENFD assessment.

Vileikyte et al. [26] previously demonstrated an association between painful and sensory symptoms in the feet and depressive symptoms in individuals with diabetes. In our study, we also show that people with depression exhibited statistically significantly higher scores for foot pain, tingling, weakness, ataxia, upper limb symptoms, and overall symptom burden compared with those without depression. The observed association between depressive symptom burden, painful neuropathy, and socioeconomic disadvantage likely reflects a complex interplay of behavioural, metabolic, and psychosocial factors. Individuals from lower socioeconomic backgrounds may experience greater chronic stress, reduced access to healthcare resources, and challenges in diabetes self-management, all of which may contribute to both psychological distress and heightened symptom perception [43]. In addition, depression may influence pain perception [44,45] and central pain modulation [46], potentially amplifying neuropathic symptom reporting. However, given the cross-sectional design, these relationships remain associative and causal pathways cannot be established.

Depression has further been linked with metabolic syndrome, characterised by hyperglycaemia, dyslipidaemia, and central obesity [23]. While Lustman et al. confirmed an association between depression and hyperglycaemia, the causal direction remains unclear [47]. Additionally, a synergistic effect was found between diabetes and depression, with an increased risk of both micro-and macro-vascular complications [48]. In our cohort, individuals with depression had higher BMI and HbA1c compared with individuals without depression, and these are established risk factors for diabetic neuropathy [33,49] and corneal nerve damage [32]. Engum et al. found no significant association between hyperglycaemia and depression in a mixed cohort of patients with type 1 and type 2 diabetes [50]. Although large population-based meta-analyses have consistently demonstrated a higher prevalence of depression in females compared with males [51], we did not observe a statistically significant sex difference in this cohort. This may reflect the modest sample size, the clinical characteristics of individuals attending specialist diabetes clinics, or differences in assessment methodology. It is also possible that the chronic disease burden associated with diabetes attenuates the typical sex disparity observed in the general population, as ongoing metabolic and complication-related stressors may influence psychological vulnerability across both sexes.

SES may influence glycaemic control, with lower SES being associated with a higher HbA1c [19]. Individuals with lower SES are at increased risk of developing depression [9,20,21], T2DM and obesity, which are often linked to adverse health behaviours [20]. HbA1c was significantly correlated with both socioeconomic indicators (SEC and IMD) and depressive symptom burden (SF-36 and VAS score) in our cohort, suggesting glycaemic control may lie on the pathway linking socioeconomic disadvantage and depression with neuropathic burden. However, due to the cross-sectional nature of the study, temporality cannot be established, and mediation cannot be formally inferred.

The absence of an observed association between SES and CCM parameters in this study may reflect the use of area-level socioeconomic indicators and the multifactorial nature of neuropathy, rather than a true lack of relationship. In our cohort, the HADS and SF-36 scores correlated with all four socioeconomic categories (SEC, IMD, MHI and LOQ). Obese individuals with T2DM residing in socioeconomically deprived neighbourhoods experience significantly poorer physical health and mental wellbeing [52]. Lower educational attainment has been associated with an increased risk of T2DM, and there is some evidence that paternal occupation and education level may influence the development of T2DM in women [53]. Ranjan et al. showed, patients with low SES had more severe symptoms of diabetes, comorbid depression and lower life satisfaction and were more likely to have common mental health disorders [54]. Poor glycaemic control has been correlated with anxiety, depression, hostility, and vulnerability [55]. In the present study, we demonestrated in our cohort of T1DM and T2DM patients that HbA1c correlated with the SF-36 score. Patients with depression and diabetes tend to have lower educational attainment [50,56], reduced income [56], and higher rates of obesity [50], findings that are largely reflected in our study population.

Living in a neighbourhood with a higher level of socioeconomic deprivation has been linked to a greater likelihood of developing symptomatic DPN in patients with T1DM [12] and more pain in patients with T2DM [13]. Furthermore, patients with the lowest SES are at the greatest risk of developing diabetes-related complications [14] and yet are least likely to access adequate healthcare services [12]. Indeed, in our study cohort, individuals with depression had significantly higher indices of multiple deprivation, with fewer patients having obtained higher educational qualifications, a lower average household income, and worse overall socioeconomic classification. Higher educational attainment and SES serve as protective factors against depression [9], and SES scores and education level differed significantly between patients with and without depression in our study.

### Limitations

This study is cross-sectional in design, providing only a snapshot of patients’ health and wellbeing at a single point in time, which limits causal inferences. Depression was categorised as a binary variable (with depression vs. without depression), without stratification by severity, which may have overlooked dose-dependent effects.

Additionally, this was a single-centre study with a relatively small sample size and limited ethnic and sociodemographic diversity, which may restrict the generalisability of the findings to broader populations. As participants were recruited from routine outpatient clinics, selection bias cannot be excluded.

Socioeconomic status was derived from area-level data rather than individual-level measures and may therefore be subject to ecological misclassification. Differences in BMI and HbA1c between groups were not adjusted for, as the primary aim of this study was exploratory and descriptive rather than determining independent effects. Given the cross-sectional design, it is not possible to distinguish whether variables such as glycaemic control act as confounders or lie on the causal pathway, and residual confounding cannot be excluded.

Future longitudinal studies incorporating causal modelling approaches are needed to clarify the temporal relationships between socioeconomic status, depressive symptomatology, and neuropathy progression, and to determine whether these factors independently or jointly contribute to small fibre decline over time in patients with type 1 and type 2 diabetes.

## 5. Conclusions

In this study, individuals with depression had significantly more neuropathic symptoms and more pronounced small fibre damage as indicated by a lower corneal nerve fibre density and corneal nerve fibre length. They also had higher BMI and HbA1c levels, indicating poorer metabolic control, which is a recognised risk factor for diabetic neuropathy. Our findings also highlight the association between SES, depression and diabetes, which may reflect disparities in healthcare access, resources, and support. These findings suggest potential implications for more integrated approaches that consider mental health and social determinants of health in the management of diabetes and its complications. Longitudinal studies are warranted to evaluate whether improvements in SES or targeted psychosocial interventions are associated with differences in the trajectory of depression and diabetic peripheral neuropathy.

## Figures and Tables

**Table 1:** Demographic data for patients with and without depression.

	Patients with Depression	Patients Without Depression	*p* Value
**Number of Patients**	60	71	-
**Age (years)**	59.98 ± 12.39	63.84 ± 10.32	0.060
**Duration of Diabetes (years)**	17.98 ± 14.25	20.58 ± 18.11	0.872
**DPN present (%)**	26.7	26.8	0.990
**Painful DPN present (%)**	23.3	7.0	<0.001
**Pain Medication (%)**	38.3	11.3	<0.001
**Gender (Sex) F-M%**	43.4–56.7	36.6–63.4	0.438
**Type of Diabetes** **(Type 1%/Type 2%)**	24.1–75.9	33.8–66.2	0.234
**Ethnicity** **(Caucasian%—Non-Caucasian%)**	76.7–23.3	94.4–5.6	0.003
**BMI (kg/m^2^)**	32.85 ± 9.72	29.07 ± 4.76	0.004
**HbA1c (mmol/mol)**	59.74 ± 12.63	53.22 ± 15.07	0.001
**Chol (mmol/L)**	4.12 ± 0.89	3.90 ± 0.74	0.190
**HDL (mmol/L)**	1.32 ± 0.45	1.49 ± 0.46	0.085
**Trig (mmol/L)**	1.72 ± 0.94	1.40 ± 0.79	0.063
**LDL (mmol/L)**	2.04 ± 0.76	1.77 ± 0.58	0.069

DPN—diabetic peripheral neuropathy, F—female, M—male, BMI—body mass index, HbA1c—haemoglobin A1c, Chol—cholesterol, HDL—high-density lipoprotein, Trig—triglycerides, LDL—low-density lipoprotein.

**Table 2:** Results of neuropathy assessments: signs and symptoms in patients with and without depression.

	Patients with Depression	Patients Without Depression	*p* Value
**CNFD (no./mm^2^)**	20.49 ± 7.55	24.16 ± 5.68	0.002
**CNBD (no./mm^2^)**	47.66 ± 29.69	56.75 ± 30.90	0.091
**CNFL (mm/mm^2^)**	20.06 ± 6.98	22.95 ± 6.22	0.014
**IENFD (no./mm^2^)**	2.05 ± 1.42	3.91 ± 2.69	0.223
**NDS (0–10)**	4.55 ± 3.11	4.25 ± 3.32	0.534
**Sural Nerve Latency (ms)**	3.26 ± 0.33	3.34 ± 0.47	0.687
**Sural Nerve Amplitude (uV)**	9.14 ± 7.20	7.63 ± 5.80	0.228
**Sural Velocity (m/s)**	43.38 ± 4.36	42.71 ± 5.72	0.499
**CPT (°C)**	22.50 ± 8.47	26.34 ± 3.08	0.007
**WPT (°C)**	41.99 ± 4.95	40.66 ± 3.93	0.105
**Sensory Score**	2.18 ± 3.21	1.69 ± 2.90	0.234
**DB-HRV (bpm)**	14.65 ± 10.04	16.92 ± 9.58	0.115
**VPT (V)**	18.92 ± 11.10	18.59 ± 12.77	0.504

CNFD—corneal nerve fibre density, CNBD—corneal nerve branch density, CNFL—corneal nerve fibre length, IENFD—intraepidermal nerve fibre density, NDS—neuropathy disability score, CPT—cold perception threshold, WPT—warm perception threshold, DB-HRV—deep breathing–heart rate variability, VPT—vibration perception threshold.

## Data Availability

The data supporting the findings of this study are available from the corresponding author upon reasonable request.

## Abbreviations

The following abbreviations are used in this manuscript:

ANOVA	analysis of variance
BMI	body mass index
CCM	corneal confocal microscopy
Chol	cholesterol
CNBD	corneal nerve branch density
CNFD	corneal nerve fibre density
CNFL	corneal nerve fibre length
CPT	cold perception threshold
DB-HRV	deep breathing–heart rate variability
DPN	diabetic peripheral neuropathy
FDR	false discovery rate
HADS	Hospital Anxiety and Depression Scale
HbA1c	haemoglobin A1c
HDL	high-density lipoprotein
IENFD	intraepidermal nerve fibre density
IMD	Index of Multiple Deprivation
LDL	low-density lipoprotein
LOQ	level of qualification
MCS	Mental Component Score
MHI	mean household income
NDS	neuropathy disability score
ONS	Office for National Statistics
SD	standard deviation
SEC	socioeconomic classification
SES	socioeconomic status
SF-36	36-Item Short Form Healthy Survey Questionnaire
T1DM	type 1 diabetes mellitus
T2DM	type 2 diabetes mellitus
Trig	triglycerides
VAS	visual analogue scale
VPT	vibration perception threshold
WPT	warm perception threshold

## Appendix A

**Table A1:** Correlations between socioeconomic factors, depression, HbA1c and neuropathy assessments.

	VAS	HADS	SF-36	HbA1c
**SEC**	r = 0.419; *p* < 0.001	r = 0.421; *p* < 0.001	r = −0.337; *p* < 0.001	r = 0.250; *p* = 0.008
**IMD**	r = 0.442; *p* < 0.001	r = 0.432; *p* < 0.001	r = −0.333; *p* < 0.001	r = 0.211; *p* = 0.027
**LOQ**	r = −0.331; *p* < 0.001	r = −0.322; *p* < 0.001	r = 0.321; *p* < 0.001	-
**MHI**	r = −0.453; *p* < 0.001	r = −0.462; *p* < 0.001	r = 0.252; *p* = 0.128	-
**CNFD (no./mm^2^)**	r = −0.311; *p* < 0.001	r = −0.229; *p* = 0.009	r = 0.241; *p* = 0.006	-
**CNBD (no./mm^2^)**	r = −0.182; *p* = 0.038	-	r = 0.206; *p* = 0.019	-
**CNFL (mm/mm^2^)**	r = −0.231; *p* = 0.008	r = −0.191; *p* = 0.029	r = 0.257; *p* = 0.003	-
**NDS (0–10)**	r = 0.226; *p* = 0.009	-	-	-
**DB-HRV (bpm)**	r = −0.271; *p* = 0.004	-	-	-
**CPT (°C)**	r = −0.345; *p* < 0.001	r = −0.266; *p* = 0.002	-	-
**WPT (°C)**	r = 0.316; *p* < 0.001	-	-	-
**VPT (V)**	r = 0.198; *p* = 0.024	-	-	-
**Symptom Score**	r = 0.635; *p* < 0.001	r = 0.551; *p* < 0.001	r = −0.515; *p* < 0.001	-
**Sensory Score**	r = 0.290; *p* < 0.001	-		-
**VAS**	-	r = 0.522; *p* < 0.001	r = −0.427; *p* < 0.001	r = 0.186; *p* = 0.049
**HADS**	r = 0.522; *p* < 0.001	-	r = −0.670; *p* < 0.001	-
**SF-36**	r = −0.427; *p* < 0.001	r = −0.670; *p* < 0.001	-	r = 0.279; *p* =0.043

SEC—socioeconomic classification, IMD—Index of Multiple Deprivation, LOQ—low level of qualification, MHI—mean household income, CNFD—corneal nerve fibre density, CNBD—corneal nerve branch density, CNFL—corneal nerve fibre length, NDS—neuropathy disability score, DB-HRV—deep breathing–heart rate variability, CPT—cold perception threshold, WPT—warm perception threshold, VPT—vibration perception threshold, VAS—visual analogue scale, HADS—Hospital Anxiety and Depression Scale, SF-36—36-Item Short Form Healthy Survey Questionnaire, HbA1c—glycated haemoglobin A1c.
